# An Advanced Hall Element Array-Based Device for High-Resolution Magnetic Field Mapping

**DOI:** 10.3390/s24123773

**Published:** 2024-06-10

**Authors:** Tan Zhou, Jiangwei Cai, Xin Zhu

**Affiliations:** 1 School of Optoelectronic Science and Engineering, Soochow University, Suzhou 215000, China; 20215239001@stu.suda.edu.cn (T.Z.); 20215239056@stu.suda.edu.cn (J.C.); 2 Suzhou Matrix Semiconductor Ltd., Suzhou 215000, China

**Keywords:** Hall sensor array, magnetic camera, nondestructive testing, signal processing

## Abstract

The precise mapping of magnetic fields emitted by various objects holds critical importance in the fabrication of industrial products. To meet this requirement, this study introduces an advanced magnetic detection device boasting high spatial resolution. The device’s sensor, an array comprising 256 unpackaged gallium arsenide (GaAs) Hall elements arranged in a 16 × 16 matrix, spans an effective area of 19.2 mm × 19.2 mm. The design maintains a 1.2 mm separation between adjacent elements. For enhanced resolution, the probe scans the sample via a motorized rail system capable of executing specialized movement patterns. A support structure incorporated into the probe minimizes the measurement distance to below 0.5 mm, thereby amplifying the magnetic signal and mitigating errors from nonparallel probe–sample alignment. The accompanying interactive software utilizes cubic spline interpolation to transform magnetic readings into detailed two- and three-dimensional magnetic field distribution maps, signifying field strength and polarity through variations in color intensity and amplitude sign. The device’s efficacy in accurately mapping surface magnetic field distributions of magnetic and magnetized materials was corroborated through tests on three distinct samples: a neodymium–iron–boron magnet, the circular magnetic array from a smartphone, and a magnetized 430 steel plate. These tests, focused on imaging quality and magnetic field characterization, underscore the device’s proficiency in nondestructive magnetic field analysis.

## 1. Introduction

In a manner akin to optical detection devices that capture reflected light to form images, magnetic detection devices utilize the measurement of magnetic field strength from magnetic or magnetized objects to generate two- or three-dimensional magnetic field distribution maps. Hall-effect-based magnetic sensors are prevalently employed in this domain, owing to their notable sensitivity and compact form factor [1]. A primary metric for assessing magnetic cameras’ performance is the spatial resolution of magnetic field distribution images, which hinges on factors such as the pixel center distance and the sensor arrangement.

Focusing on pixel center distance, conventional magnetic detection devices predominantly utilize packaged magnetic sensors. Chen-Wei Liang and associates [2] developed a magnetic imaging system employing a 16 × 16 quantum well Hall effect sensor array as its probe, achieving a pixel center distance of 5 mm for detecting surface anomalies in ferromagnetic materials. Andriyan Bayu Suksmono and collaborators [3] crafted a magnetic imaging system composed of anisotropic magnetoresistance triaxial magnetometers, adept at measuring ferromagnetic objects’ magnetic field distributions with a pixel center distance exceeding 1 cm. Jinlong Dong and team [4] innovated a Hall effect sensor array-based magnetic field measurement system with a 3.5 mm pixel center distance. This system enables the reconstruction of electric arc current density from magnetic field data, facilitating the analysis of arc dynamics through the magnetic field inverse problem.

Regarding sensor arrangement, various configurations exist, including single Hall placement, linear Hall arrangement, Hall array, and circular Hall configuration. Nandita Saha and colleagues [5] employed a single Hall element probe, positioned vertically over an iPhone 13 Pro, to scan and delineate the internal circular magnetic array, speaker, and camera modules. The resultant magnetic field distribution images were instrumental in examining the interference of stray magnetic fields from electronic devices on medical equipment, thereby assessing potential risks. Jinyi Lee and team [6,7] devised a magnetic imaging device with a linear array of 64 InSb Hall sensors, suitable for identifying wear and fissures in train wheels. J.Z. Wang [8] introduced an 8 × 8 Hall sensor array for magnetic flux density detection, corroborated through comparative software simulation analyses. Jungmin Kim and others [9] arrayed 45 Hall sensors in a circular pattern to detect radial magnetic field components at pipeline defects, contributing to the quantitative assessment of these flaws.

Measurement distance emerges as a pivotal factor impacting imaging quality in magnetic field detection. The rapid attenuation of magnetic field strength with increasing distance necessitates the proximity of the sensor probe to the test object, maintaining a consistent measurement gap [10]. Excessive or inconsistent measurement distances can lead to the loss of critical magnetic field data and introduce unnecessary errors, thus diminishing the reliability of the magnetic detection device. In [11], a one-dimensional linear array Hall sensor, with a measurement distance of 10 mm, was deployed to ascertain the shape and magnetic field distribution of ferromagnetic objects, despite the presence of obstructions. The findings suggested that the magnetic field distribution map could approximately delineate the object’s contour and magnetic characteristics. Similarly, in another research [2], a two-dimensional array magnetic camera was constrained to a 4.3 mm measurement distance due to geometric limitations when assessing the magnetic field distribution of a sample.

Moreover, magnetic imaging finds significant application in nondestructive testing, a method that enables the detection of surface cracks or internal defects in materials without inflicting damage [12]. Common electromagnetic techniques in this realm include eddy current testing [13,14], magnetic particle inspection, and magnetic flux leakage detection [15,16,17]. Specifically for ferromagnetic materials, nondestructive evaluation employing magnetic flux leakage detection is advantageous. This technique involves magnetizing the sample and subsequently detecting the magnetic leakage field distribution on its surface, allowing for both qualitative and quantitative defect analysis [18].

In this study, we developed a Hall element array-based magnetic detection device designed for examining the surface magnetic distribution of magnetic and ferromagnetic objects. To validate the device’s reliability, we initially tested a simple-shaped neodymium–iron–boron magnet and then proceeded to assess the circular magnetic array within a smartphone as an example of an industrial product. For nondestructive testing applications, a 430 steel plate with artificially induced defects was utilized as a test sample. Furthermore, to enhance measurement accuracy, we employed wear-resistant ceramic plates as a support structure to mitigate measurement discrepancies. Additionally, the specialized movement path of the rail system significantly augmented the spatial resolution of our device.

## 2. Configuration of the Magnetic Detection Apparatus

The magnetic detection apparatus delineated herein comprises four principal components: a sensor probe constituted by an array of Hall elements, a signal conditioning readout circuit board, an *X*-*Y* axis electromechanical scanning rail, and software for interactive magnetic field visualization.

### 2.1. Array of Hall Elements

**Figure 1:**
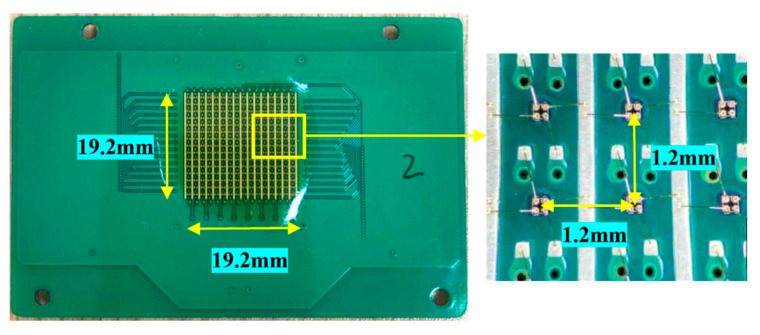
Hall array board physical drawing.

In comparison to conventional silicon-based Hall elements, gallium arsenide (GaAs) Hall elements exhibit superior sensitivity, exceeding that of their silicon counterparts by over eightfold [19], alongside enhanced temperature performance characteristics [20]. This renders them particularly suitable for applications in linear sensing. The spatial resolution of pixels in packaged Hall sensors is limited by enclosure constraints to the millimeter range. Utilizing an unpackaged array of Hall elements as the sensing probe allows for the strategic placement of supply and output pads and vias on either side, thereby not compromising the vertical separation, and achieving interelement spacing on the order of hundreds of micrometers. The deployment of Hall elements in an array, necessitating individual mounting onto designated PCB zones with careful consideration for surrounding gaps, yields a pixel center-to-center distance that surpasses that observed in linear configurations. Nonetheless, when taking into account measurement duration and scope, the strategic advantage lies with the array configuration. It is anticipated that advancements in PCB precision manufacturing techniques may facilitate a reduction in the spacing between elements within the array. In this study, 256 unpackaged GaAs Hall elements were organized into a 16 × 16 matrix, as depicted in Figure 1, positioned on a PCB and interconnected via metal leads to pads, with the entire PCB dimensioned at 7.8 cm by 5.2 cm. Microscopic examination reveals the layout of each Hall element, where the distance between adjacent elements is noted to be 1.2 mm, encompassing a total area of 19.2 mm by 19.2 mm. A parallel power supply configuration is employed for each column of 16 Hall elements, regulated through voltage biasing.

### 2.2. Signal Readout Circuitry

**Figure 2:**
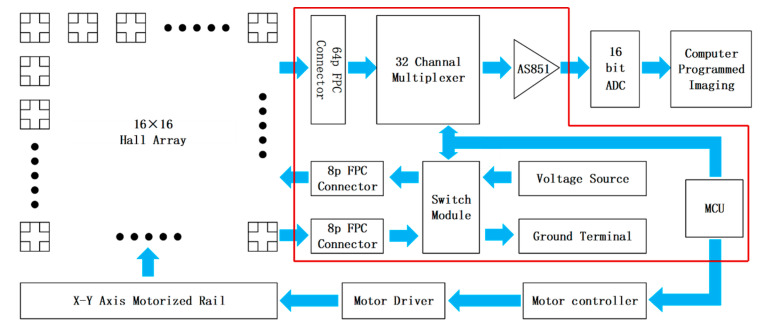
Block diagram of the overall system of the magnetic detection device.

Illustrated in Figure 2, the system schematic of the magnetic detection apparatus, the readout circuitry is demarcated by a red border. The circuit consists mainly of FPC connectors, multiplexers, switching modules, programmable gain amplifiers, and a microcontroller. Its chief role is to select and amplify the Hall voltage signals, and convey these signals to the data acquisition system, while the biasing condition of the Hall element array is managed by the switching module. The array is segmented into 16 linear sensor regions, each 16 × 1, with the signals from each column of Hall elements relayed to the readout circuit board’s 32-channel multiplexer via the FPC connector. Upon receiving a control signal from the microcontroller, the multiplexer transmits the relevant Hall voltage signal to the amplifier, transforming the differential signal detected by the Hall elements into a single-ended output. The amplifier, designed as a programmable linear amplifier, allows for presetting of the gain to ensure rapid response in high-bandwidth scenarios. Concurrently, the microcontroller dispatches a control signal to the switching module, orchestrating the activation of the power and ground terminals for each column of Hall elements horizontally, thereby mitigating signal interference. After signal selection and amplification, the Hall voltage signals are forwarded to a 16-bit high-speed analog-to-digital converter (ADC), operating in continuous acquisition mode, with the data continuously transferred to the computer’s memory via the USB interface. After the Helmholtz coil calibration, the Hall sensitivity was 7.1 mV/mT at 4 V bias voltage (amplifier gain was set to 50), the detection bandwidth was at 64 KHz, and there was a maximum detection of a magnetic field of 352 mT.

**Figure 3:**
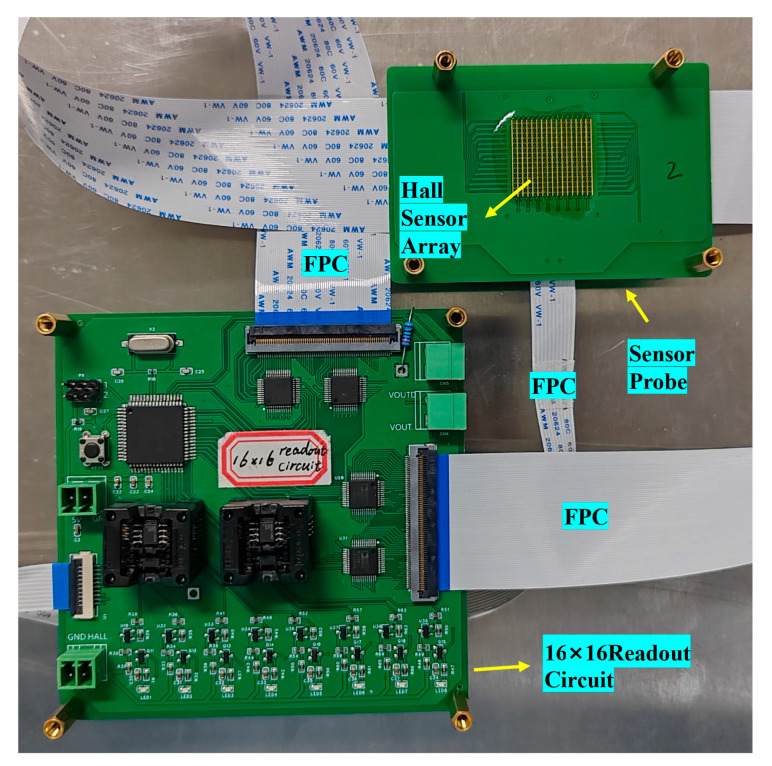
Connection of readout board and Hall array board via FPC.

Figure 3 presents the connection between the Hall element array and the readout circuit board, transmitting the Hall voltage signals and regulating the bias state through the FPC connector.

### 2.3. Electric X-Y Axis Rail

**Figure 4:**
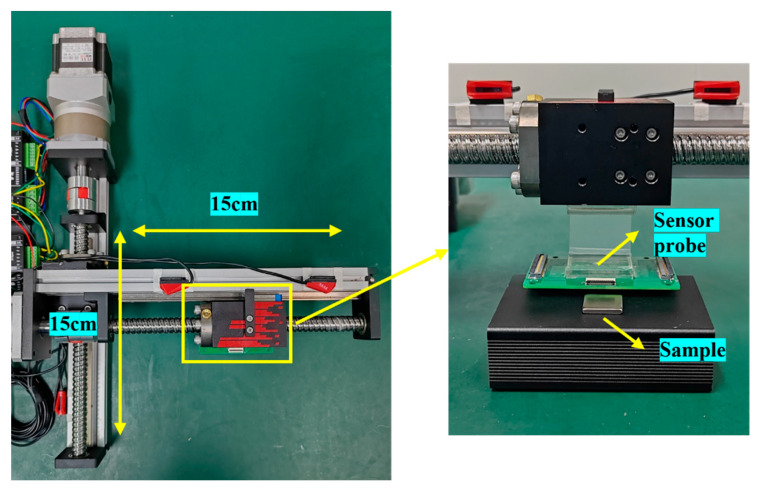
Schematic diagram of electric rail.

For measurements across a broad range, precise control over the sensor probe’s movement is imperative. Consequently, the apparatus integrates the sensor probe onto electromechanical rails for scanning detection, as depicted in Figure 4. The Hall array board is located below the electric rail and the test object is placed immediately below the Hall array board to ensure minimal attenuation of the magnetic field strength. The rail’s step angle is set at 1.8°, achieving a repeated positioning accuracy of ±10 μm and an effective scanning area of 15 cm × 15 cm, which suffices for the magnetic field examination of a wide array of magnetic objects.

### 2.4. Software Integration

**Figure 5:**
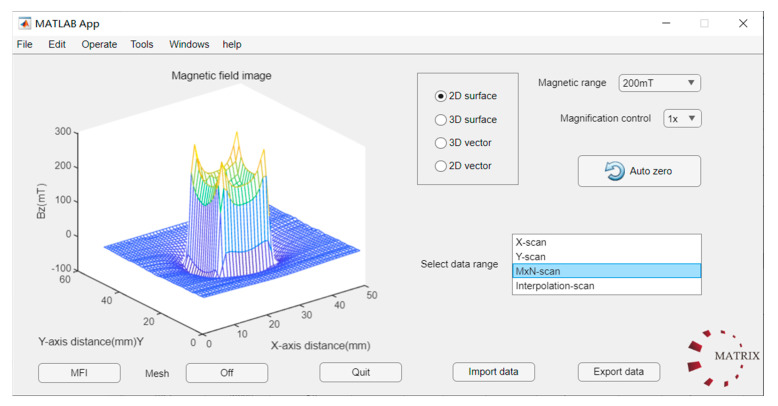
Schematic diagram of the interactive software.

For the analysis of acquired data, an interactive magnetic imaging interface utilizing MATLAB R2023a was engineered. As demonstrated in Figure 5, this interface presents the magnetic field distribution of a square magnet, before interpolation, in a three-dimensional visualization. The data’s sign conveys the magnetic field’s polarity, whereas the color intensity reflects the field’s magnitude. Users are afforded the capability to manually adjust the interpolation scanning process, selecting the data range based on various scanning trajectories of the electric rails, and applying distinct algorithms for data stitching and interpolation. Additionally, the software is equipped with calibration capabilities; by positioning the sensor probe within Helmholtz coils, discrepancies between measured and actual values can be calibrated.

**Figure 6:**
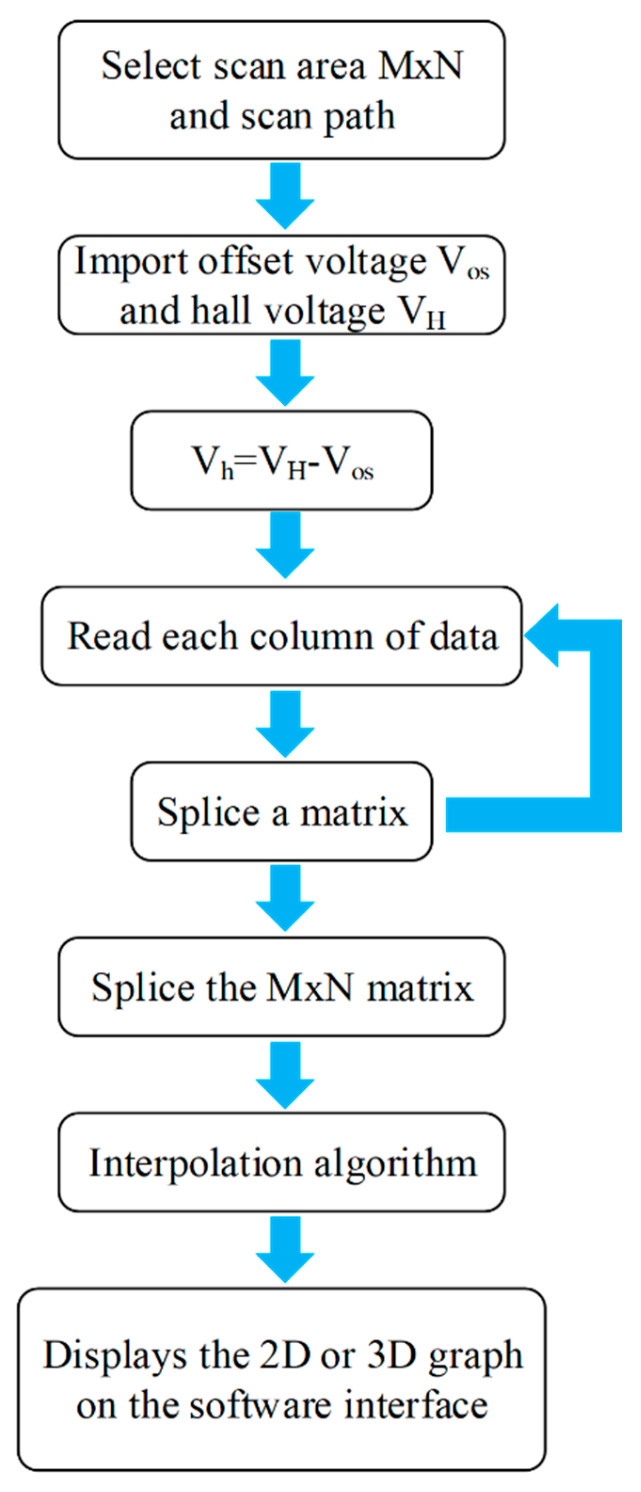
Flowchart of the program.

The procedural flowchart, depicted in Figure 6, necessitates the selection of the scanning area, denoted as M × N, and the scanning trajectory before data processing, where M and N symbolize the count of scanning zones in horizontal and vertical orientations, respectively. This choice influences the selection of stitching and interpolation algorithms. Subsequently, the software initiates the reading of each Hall element’s offset voltage V_os_ and Hall voltage V_H_, facilitating the computation of the Hall signal V_h_. Following the acquisition of all V_h_ signals within the designated scanning range, a stitching algorithm is employed to amalgamate the Hall signals obtained along the scanning path into a unified matrix. Cubic spline interpolation is utilized for this purpose. The finalized data are thereafter transformed into an image for display within the software interface, enabling visual analysis.

## 3. Scheme for Magnetic Detection

### 3.1. Interpolation Scanning

**Figure 7:**
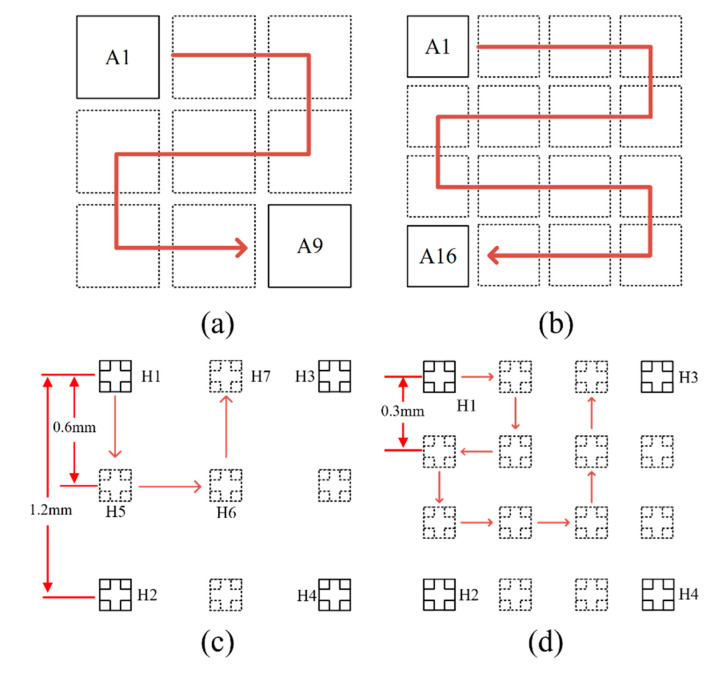
Interpolation scanning diagram (**a**) 3 × 3 scanning area; (**b**) 4 × 4 scanning area; (**c**) before interpolation scan; (**d**) after interpolating the scan.

Before initiating measurements, it is essential to establish a predefined trajectory for the electric rails’ movement. Illustrated in Figure 7a, the designated path for a 3 × 3 scanning grid, with A1 to A9 marking each respective scanning zone, encompasses a detection area of 57.6 mm × 57.6 mm; similarly, Figure 7b delineates the trajectory for a 4 × 4 scanning grid, spanning a detection area of 76.8 mm × 76.8 mm. Within the entirety of the detection zone, the inter-pixel spacing is maintained at 1.2 mm. This document proposes an innovative rail movement strategy capable of diminishing the pixel-to-pixel distance, thereby enhancing the overall resolution of the imaging process. As depicted in Figure 7c, within the A1 scanning zone, four Hall elements, H1 through H4, are positioned 1.2 mm apart. In transitioning to the subsequent scanning zone from A1, the control mechanism advances the Hall element array downward by half a pixel’s breadth, effectively repositioning H1 to H5, which facilitates the capture of intermediary magnetic field data between H1 and H2. Subsequently, the array is shifted half a pixel both rightward and upward to positions H6 and H7, respectively. This methodology, through algorithmic processing, integrates the newly acquired data points amidst the original set, halving the pixel-to-pixel distance to 0.6 mm. Altering the scanning technique, as illustrated in Figure 7d, further reduces the inter-pixel distance to one-third of its initial value, now at 0.3 mm. Employing a denser scanning protocol could potentially decrease the pixel-to-pixel distance even more, significantly refining imaging resolution, while the number of scans will be correspondingly increased, which places higher demands on the precise movement of the motorized guides, and the detection efficiency will be greatly reduced when interpolating more than twice. Comprehensive consideration of various parameters, including the dimensions of the object under examination, is advised before measurement.

### 3.2. Measuring Cycle

**Figure 8:**
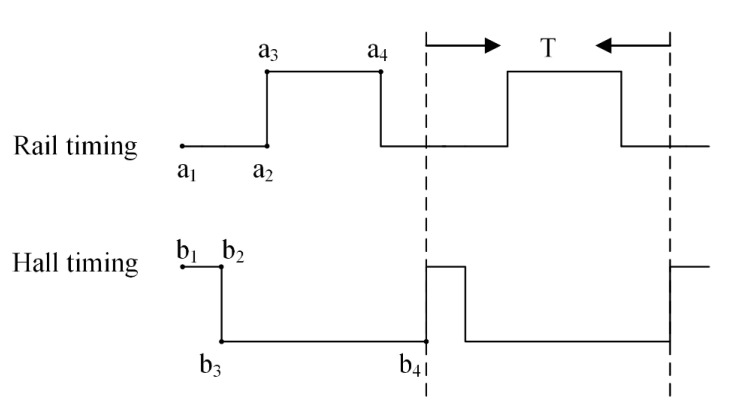
Temporal matching diagram.

It is imperative to space out the running time of the electronic rail from the duration of the Hall signals. As shown in Figure 8, the initiation of the magnetic imaging apparatus triggers a period (denoted a1 to a2) which represents the delay time of the rail. During this period, Hall voltage signals are output sequentially throughout b1 to b2. Similarly, a3a4 and b3b4 denote the travel time of the rail and the cutoff time of the switching module, respectively. For this apparatus, the operational cycle time, T, is established at 350 ms, with each sensor zone covering an area of 19.2 mm × 19.2 mm. Consequently, to scan and ascertain the magnetic field across an area of 57.6 mm × 57.6 mm necessitates eight movements, culminating in a duration of 2.8 s, whereas surveying a 76.8 mm × 76.8 mm area demands fifteen movements, accruing a total time of 5.25 s.

### 3.3. Direct Contact Measurement

**Figure 9:**
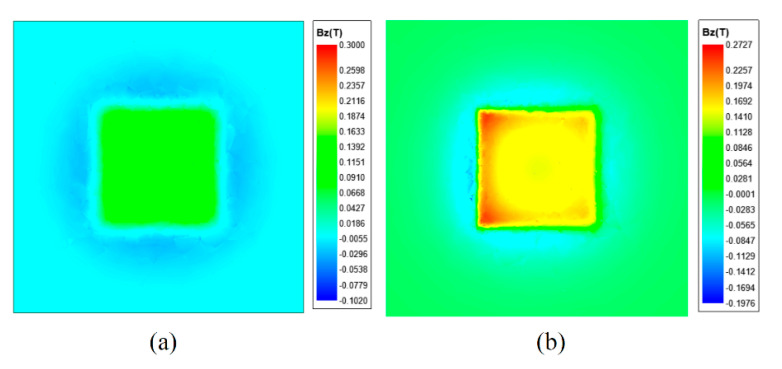
Schematic simulation of the square magnet (**a**) depicts the measurement distance between the square magnet and the sensor probe as 2 mm; (**b**) shows the square magnet not aligned parallel to the sensor probe.

To ensure the magnetic field distribution on the sample’s surface is accurately represented and the measurement data are consistently analyzed, the experimental methodology strives to minimize and standardize the distance between the sensor probe and the sample. The magnetic field distributions for a square magnet, simulated via Maxwell 2022 software, are illustrated in Figure 9a,b. As demonstrated in Figure 9a, a testing distance of 2 mm from the square magnet approximates the conventional measurement setup. At this proximity, the magnetic field strength attenuates severely, leading to blurring of the magnet edges on the image. As shown in Figure 9b, the lack of parallel alignment between the square magnet and the sensor probe introduces discrepancies in signal amplitude. When the simulation surface is angled 1° off parallel in the XY plane relative to the square magnet, a significant disparity in signal amplitude between the magnet’s left and right sides is noted, compromising the fidelity of the magnetic field distribution representation.

**Figure 10:**
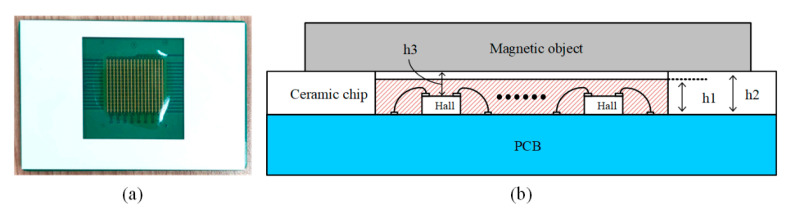
Use of a wear-resistant ceramic piece as a support structure (**a**) Hall array probe; (**b**) contact measurement schematic.

To mitigate the outlined challenges, this study introduces the incorporation of a wear-resistant ceramic piece (Figure 10a) onto the PCB as a structural support to diminish measurement inaccuracies. The ceramic sheet is cut by a laser process, and the height difference between the left and right sides is within 0.01 mm, which brings an angular deviation of less than 0.01°, much smaller than the error of noncontact measurement. Figure 10b shows that the Hall element array’s total height is denoted as h1. A grooved ceramic piece, with a thickness of h2 marginally exceeding h1, is affixed atop the PCB’s surface. This arrangement facilitates direct contact between the sensor probe and the sample through the ceramic piece, promoting contact-based measurement. The gap, h3, bridging the top surface of the Hall elements and the magnetic object, is, thus, reducible to below 0.5 mm. Employing this measurement technique amplifies the magnetic field signal and precludes inaccuracies stemming from nonparallel alignment between the sample and the sensor probe.

## 4. Experimental Results

The operational efficacy of the magnetic detection apparatus was substantiated through the examination of three distinct samples: a square neodymium–iron–boron (NdFeB) magnet, the circular magnet array situated inside the iPhone, and a flawed 430 stainless steel sheet, with all measurements executed at ambient temperature.

### 4.1. Testing Square NdFeB Magnets

**Figure 11:**
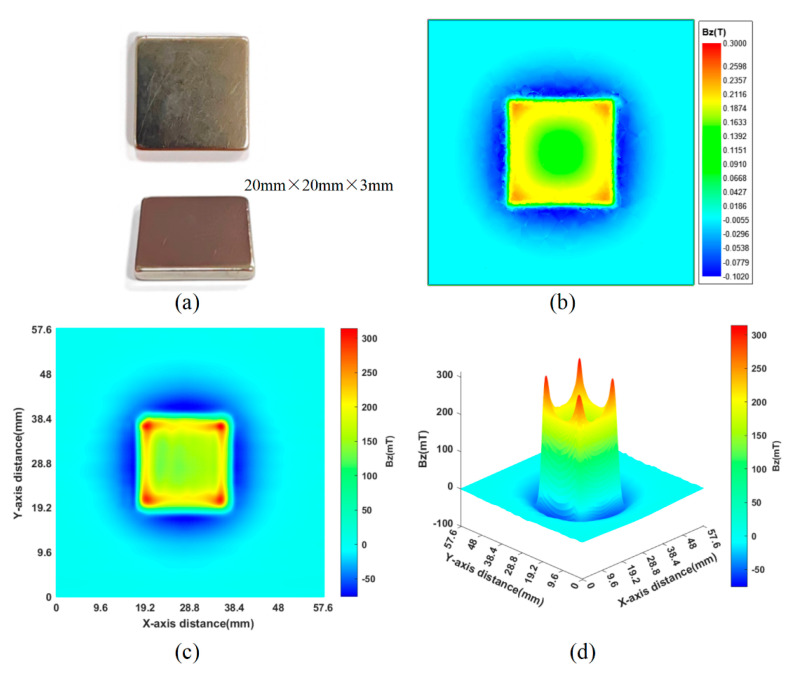
Magnetic field distribution of square NdFeB magnets: (**a**) magnet size; (**b**) Maxwell simulation results; (**c**) 2D magnetic field distribution; (**d**) 3D magnetic field distribution.

The specified dimensions of the sample, as depicted in Figure 11a, facilitated the simulation of the magnet’s magnetic field distribution. As shown in Figure 11b, the simulation plane is positioned 0.5 mm away from the surface of the magnet, and due to the effect of magnetic field convergence, it can be found that at the four corners, the magnetic field strength significantly exceeds that of the other regions on the surface. This phenomenon can be used as a basis for a preliminary judgment of the accuracy of the magnetic field measurements. Employing the 16 × 16 magnetic detection apparatus, the scanning zone was determined to be 3 × 3, with a total measurement duration of 2.8 s. Figure 11c,d show the 2D and 3D magnetic field distribution images of the samples, respectively. It is seen that the detection pattern has the magnetic field characteristics of a square magnet, verifying that the device can detect magnetic field distribution, and from the 2D measurement results, it can be found that the side length of the square magnet is 20 mm, corresponding to the shape of the magnet itself.

### 4.2. Testing the Array of Circular Magnets on the Back of a Smartphone

**Figure 12:**
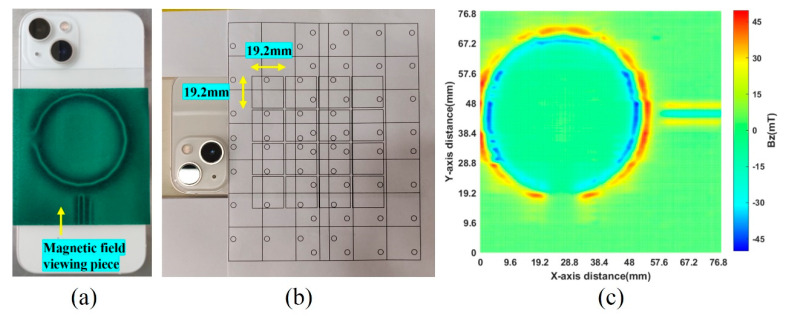
Magnetic field distribution of a circular magnet array: (**a**) placement of the magnetic field viewing piece behind the smartphone; (**b**) manual movement for measuring the circular magnet array; (**c**) measurement results of the circular magnet array.

This experiment utilized the 16 × 16 magnetic detection system to assess the circular magnet array positioned behind a smartphone. As shown in Figure 12a, with the iPhone as an example, a magnetic flux viewing film is placed on the back of the phone to identify the position of the circular magnet array. The scanning range was preset to a 4 × 4 grid (Figure 12b), and the detection was first performed using a manual movement method, with the total measurement time lasting about 5 min, and the error of the manual movement being about 3 mm. The observations, as shown in Figure 12c, disclosed discrepancies attributed to the lack of parallel alignment between the sensor probe and the smartphone’s rear surface, along with the inaccuracies inherent to manual navigation. These variances are evidenced in the imagery by the irregular amplitude of magnetic field strength and the fragmented nature of the 2D magnetic field distribution diagram.

**Figure 13:**
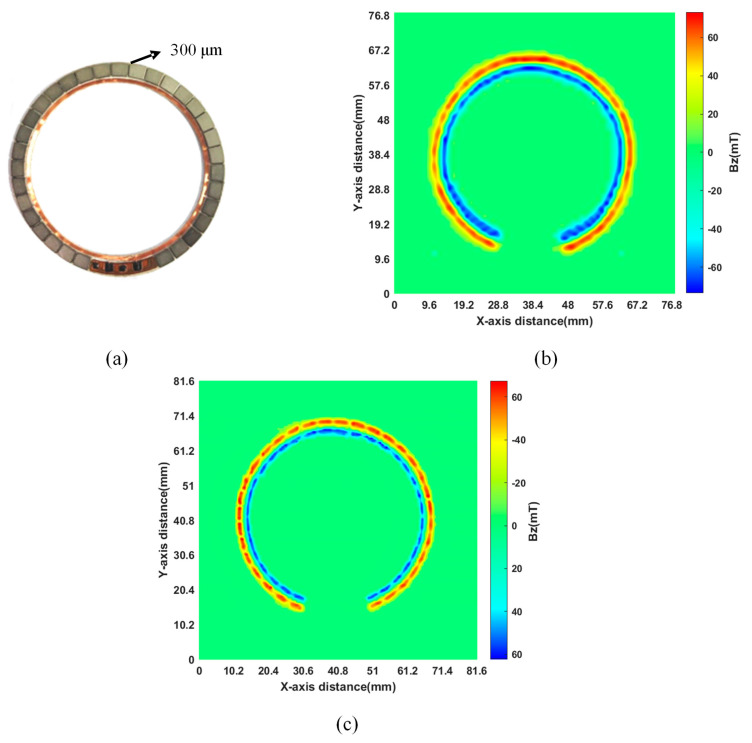
Magnetic field distribution of a circular magnet array: (**a**) circular magnet array; (**b**) measurement results with a pixel center distance of 1.2 mm; (**c**) measurement results with a pixel center distance of 0.6 mm.

To ascertain the efficacy of enhancing resolution, interpolation scanning utilizing electric rails was conducted to remove the smartphone case and reassess it. Illustrated in Figure 13a, a configuration of 32 trapezoidal magnets is arrayed circumferentially, maintaining a separation of 300 μm between adjacent units. Gaussmeter assessments of the magnet surfaces disclosed a peak magnetic field intensity of 60 mT. Figure 13b shows that initial measurements without interpolation scanning aligned with the gaussmeter’s magnetic field strength amplitude readings. Nevertheless, the 1.2 mm pixel center distance at that juncture rendered the separations within the circular magnet array indistinct in the 2D magnetic field visualization. Subsequent application of interpolation scanning via electric rails diminished the pixel center distance to 0.6 mm, thereby enhancing resolution twofold. The observations, presented in Figure 13c, reveal the discernibility of gaps within the circular magnet array in the 2D magnetic field schematic, mirroring the actual arrangement.

### 4.3. Testing 430 Stainless Steel Sheet for Surface Defects

**Figure 14:**
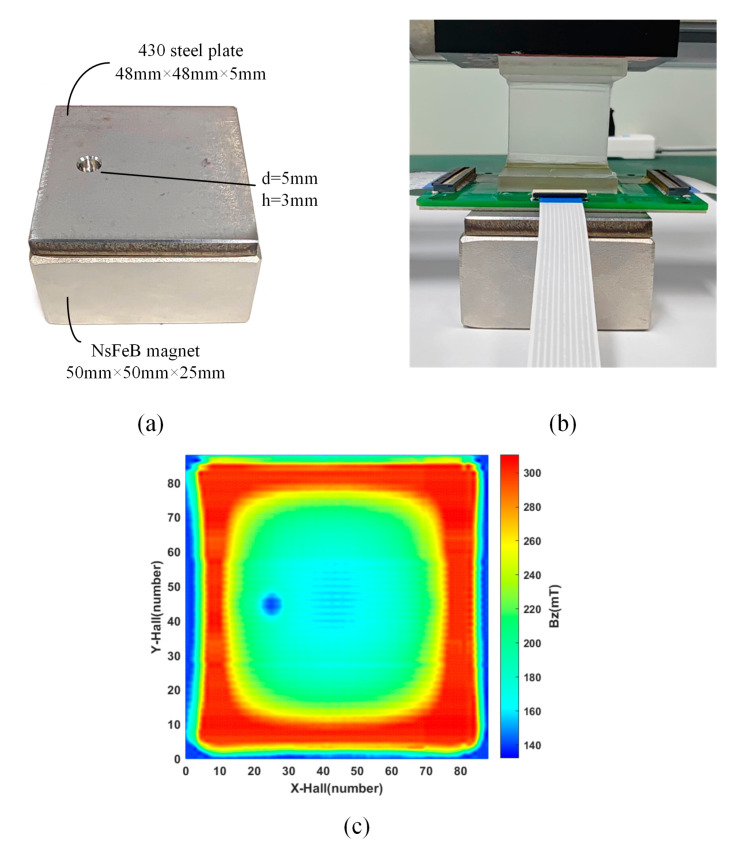
Magnetic leakage detection: (**a**) the sample with a defect; (**b**) schematic of contact measurement; (**c**) detection results for the 430 stainless steel plate.

Nondestructive evaluation (NDE) represents a critical methodology employed in industrial product examination, development, fabrication, and quality assurance. One such NDE technique, magnetic flux leakage (MFL) testing, leverages electromagnetic phenomena to identify surface anomalies in ferrous materials. As shown in Figure 14a, the 430 stainless steel plate measures 48 mm × 48 mm × 5 mm and has a circular defect with a diameter of 5 mm and a depth of 3 mm. The size of the NdFeB magnet is 50 mm × 50 mm × 25 mm. Gaussmeter evaluations revealed the magnet’s surface to exhibit a maximal magnetic field intensity of 400 mT. Positioned beneath the 430 steel plate, the NdFeB magnet served to magnetize the specimen. As outlined in Figure 14b, the experimental arrangement required direct interfacing of the sensor probe with the plate’s upper surface, surveying a 3 × 3 grid. By applying interpolation scanning, the pixel-to-pixel distance was reduced to 0.6 mm, and the results are shown in Figure 14c, where the contours of the circular defects are accurately observed in the two-dimensional magnetic field distribution image. These findings affirm the proficiency of the 16 × 16 magnetic imaging apparatus in executing high-definition nondestructive evaluations, showcasing its applicability to practical diagnostic contexts.

**Figure 15:**
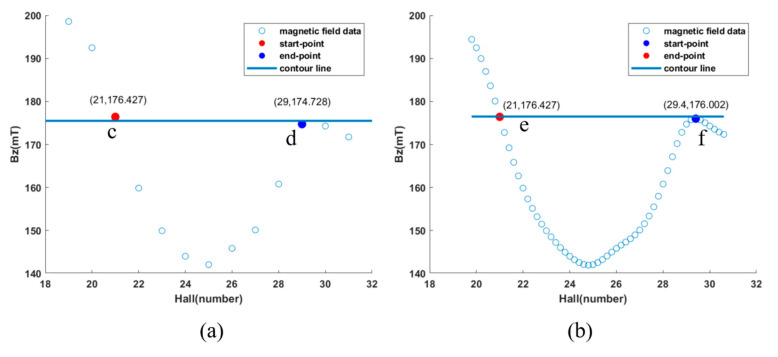
Defect size inspection: (**a**) before cubic spline interpolation; (**b**) following cubic spline interpolation.

To assess the correlation between the size of the leakage magnetic signal depicted in the magnetic field distribution diagram and the actual dimensions of the circular defect, the three-dimensional renderings of the defect zone were enlarged and scrutinized. Figure 15a,b display the visualizations before and following cubic spline interpolation, respectively.

The calculation of Formula (1) delineates the horizontal span between two measurement points that exhibit the nearest amplitude values, using the central axis of the circular defect as a reference point.

L = D × (Xright − Xleft)(1)

Here, L represents the horizontal distance, meaning the diameter of the circular defect; D represents the pixel center distance, fixed at 0.6 mm; Xright and Xleft denote the X-coordinate positions of these endpoints, reflecting the horizontal count of Hall elements. As indicated in Figure 15a, with markers at points c and d, L computes to 4.8 mm, exhibiting a 4% deviation from the true diameter of the circular defect. The application of cubic spline interpolation minimizes this discrepancy, as evidenced by markers at points e and f, with L recalculated to 5.04 mm, now showing a marginal 0.8% deviation from the actual diameter. This demonstrates the potential of a 16 × 16 magnetic imaging system to quantitatively classify defect sizes based on magnetic field distribution images. Future integrations might involve employing machine learning algorithms for the automated quantification of defect dimensions.

## 5. Conclusions

Reflecting upon the outcomes derived from the trio of cases examined herein, the magnetic detection apparatus devised in the course of this study demonstrates proficiency in mapping the magnetic field distributions of both magnetic and magnetized entities. The alignment of measurement profiles with the physical contours of the objects, coupled with the close mirroring of amplitude values to those recorded via Gaussmeter, proved the accuracy of the magnetic field measurements. Subsequent interpolated scans using motorized guides also significantly improved the resolution, allowing for the identification of even finer details. In addition, in the field of nondestructive testing, the instrument can identify surface anomalies on magnetized specimens and measure the dimensions of defects, allowing it to be developed for use in conjunction with practical needs. Anticipated avenues for forthcoming investigative endeavors encompass:

1. Deployment of flexible PCB substrates to facilitate assessments over contoured surfaces;
2. Minimization of inter-pixel spacings within the Hall sensor matrix via advanced PCB fabrication techniques;
3. Application of machine learning paradigms to automate the quantification of defect dimensions during nondestructive evaluations;
4. Circuitry improvements designed to reduce signal interference and thereby improve the overall quality of the imaging output.

## Data Availability

Data are contained within the article.
